# The Complexity of Zoonotic Filariasis Episystem and Its Consequences: A Multidisciplinary View

**DOI:** 10.1155/2017/6436130

**Published:** 2017-05-31

**Authors:** Fernando Simón, Javier González-Miguel, Alicia Diosdado, Paula Josefina Gómez, Rodrigo Morchón, Vladimir Kartashev

**Affiliations:** ^1^Laboratory of Parasitology, Faculty of Pharmacy, University of Salamanca, Salamanca, Spain; ^2^Institute of Natural Resources and Agrobiology of Salamanca (IRNASA-CSIC), Salamanca, Spain; ^3^Department of Infectious Diseases, Rostov State Medical University, Rostov-na-Donu, Russia

## Abstract

Vector-borne transmitted helminthic zoonosis affects the health and economy of both developing and developed countries. The concept of episystem includes the set of biological, environmental, and epidemiological elements of these diseases in defined geographic and temporal scales. Dirofilariasis caused by different species of the genus* Dirofilaria* is a disease affecting domestic and wild canines and felines and man, transmitted by different species of culicid mosquitoes. This complexity is increased because* Dirofilaria* species harbor intracellular symbiont* Wolbachia* bacteriae, which play a key role in the embryogenesis and development of dirofilariae and in the inflammatory pathology of the disease. In addition, the vector transmission makes the dirofilariasis susceptible to the influence of the climate and its variations. The present review addresses the analysis of dirofilariasis from the point of view of the episystem, analyzing the complex network of interactions established between biological components, climate, and factors related to human activity, as well as the different problems they pose. The progress of knowledge on human and animal dirofilariasis is largely due to the multidisciplinary approach. Nevertheless, different aspects of the disease need to continue being investigated and cooperation between countries and specialists involved should be intensified.

## 1. Introduction

Vector-borne zoonotic transmitted diseases cause deaths and economic losses in human and domestic animal populations around the world, affecting seriously the social and economic development of many countries [1, 2]. Dirofilariasis is a helminthic zoonosis caused by filarial species of the genus* Dirofilaria* transmitted by hematophagous dipterans that primarily parasitize domestic dogs, cats, and other species of wild mammals [3]. Although some* Dirofilaria* species cause relatively benign processes, others such as* D. immitis*, responsible for cardiopulmonary dirofilariasis, pose a risk to the life of affected animals, being considered the most important parasitic disease of dogs in the USA [4]. Since many of the vector species feed indistinctly on animal reservoirs and on humans, where animal dirofilariasis exists, human infections occur [5]. Human dirofilariasis has historically been considered a minor accidental disease, but the dramatic increase in cases of some clinical variants in recent years has made it nowadays considered an emerging disease in Europe [6–8]. Additionally, its clinical importance is increasing due to the severity of some cases [9, 10]. The concept of vector-borne disease episystem includes the set of biological and environmental elements, as well as the epidemiological aspects of these diseases in defined geographic and temporal scales. Because its intrinsic nature, the episystems are in constant change, reflecting the adaptations of all its components to new situations [11]. An analysis of dirofilariasis from the point of view of the episystem allows us to understand its complexity and the consequences that this implies, encompassing in all its amplitude the need for a multidisciplinary approach.

## 2. The Episystem of Dirofilariasis

### 2.1. Biological Complexity

**Figure 1:**
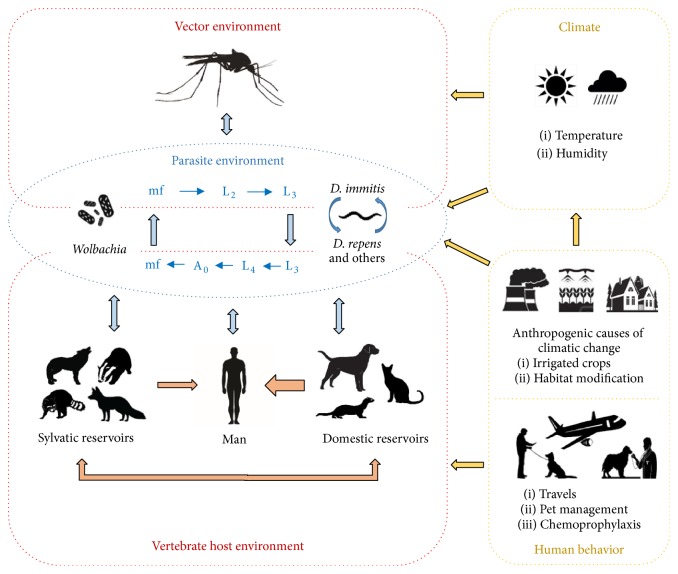
The episystem of dirofilariasis. Main interactions among organisms involved, climate, and human-derived behavior factors.

**Figure 2:**
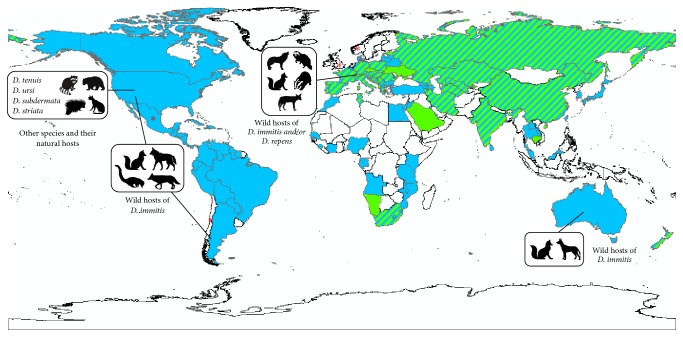
Geographic distribution of the different species of* Dirofilaria* in the animal hosts in the world.* D. immitis* in pets (blue);* D. repens* in pets (green);* D. immitis* and* D. repens* in pets (striped); without information (white); (*∗*) sporadic subcutaneous infections.

The biological component of the episystem of dirofilariasis is extremely complex as* Dirofilaria* spp. parasitize a wide range of vertebrate species and vectors (Figure 1), all of which have their own level of adaptation. During a blood meal, vectors deposit a hemolymph on the wound, which carries infective third-larval stage (L3) of* Dirofilaria* that penetrates the host's skin by their own. L3 successively molt to L4 and adults, which are located in the circulatory system and subcutaneous/ocular and many other tissues, depending on the species. In canines, female worms release microfilariae (mf) into the bloodstream, from where they are ingested by vectors during the blood meal, becoming infective after 2 additional molts. Nevertheless, some infected dogs present occult or amicrofilaremic infections. In felids and other hosts, microfilaremia is either not present or transitory and present at low levels, while in humans the worms do not usually reach maturity [6]. One or several* Dirofilaria* species may be present, depending on the area being considered (Figure 2), of which* D. immitis* and* D. repens* are considered the most significant owing to their wide distribution and clinical importance. On the other hand, the actual prevalence of species such as* D. tenuis*,* D. ursi*,* D. subdermata*, and* D. striata* in their natural wild hosts is not known [12, 13]. It should also be taken into account that L3, L4, adults, and mf can coexist in vertebrate hosts and can present different anatomical localizations, immune repertoires, and survival strategies; thus each infected host actually faces several organisms with different biological capabilities. Furthermore, all of the developmental stages of* Dirofilaria* harbor intracellular symbiotic* Wolbachia* bacteriae that are essential for the molting and embryogenesis of the worms [14]. Filarial death causes the release of the bacteriae, which establish a direct relationship with the host, a key fact in the progression of dirofilariasis [6].

Pets, such as dogs, cats, and ferrets, and a wide range of wild carnivorous species are hosts for* D. immitis* and/or* D. repens* [3, 6, 15]. The other species exclusively parasitize wild animals like raccoons, porcupines, bears, and wild felids [12].* D. immitis* and* D. repens* show a high prevalence in pets and wild reservoirs and have a high zoonotic potential [6, 16], while those species that only infect wild reservoirs are less frequently or sporadically reported in humans [12].* Dirofilaria* species are transmitted by culicid mosquitoes, except* D. ursi* which uses* Simulium* spp. as vectors. Different species of the genera* Aedes*,* Anopheles, Culex*,* Culiseta, Mansonia,* and* Armigeres* have been implicated in the transmission of* D. immitis* and* D. repens* [17].

### 2.2. Extrinsic Factors

Climate and human activity influence the biological life cycle of* Dirofilaria* spp. (Figure 1). Given that mosquitoes are ectothermic organisms with a life cycle linked to water, climatic factors, mainly temperature and precipitation/humidity, affect their development, population density, period of activity, and species diversity. Also, the development of L3 larvae depends on environmental temperature (extrinsic incubation), which includes a period of 8 to 20 days with temperatures ranging from 22° to 30°C. Below 14°C development arrests transiently until temperature reaches the threshold again [6]. On the other hand, urbanistic demands, the construction of irrigation systems, and water storage areas, the use, or not, of chemoprophylaxis and the transport and import of pets between endemic and nonendemic areas contribute to environmental changes and to the introduction of infected reservoirs in nonendemic areas, changing prevalence [6, 18–21]. The hunting pressure on potential wild reservoirs of* Dirofilaria* spp. and the anthropogenic influence on the natural environment can have epidemiological consequences, affecting the circulation of filariae between wild reservoirs, pets, and humans [15].

### 2.3. Interactions

The interactions established between* Dirofilaria* spp. and their vertebrate hosts, between developing larvae and vectors, between the different species of* Dirofilaria,* and between the developmental stages within the same species all contribute to the regulation of the parasite population and as a consequence to its transmission. In dogs, live* D. immitis* worms stimulate a permissive humoral Th2 response that has been associated with microfilaremic infections. When adults and mf die, the released* Wolbachia* bacteriae activate a change towards a Th1-type response, which in addition to causing inflammation and deterioration of the vascular environment is also associated with suppression of mfs [22, 23]. Moreover, in general, dogs are able to maintain the adult population at levels compatible with their own survival, eliminating a significant part of the L3 larvae acquired by reinfections [24]. Like other parasites* D. immitis* manipulates the immune system and various physiological processes of the host for their own benefit [25]. The elimination of significant amounts of surface antigens by the L3 larvae, the presence of proteases that lyse antibodies on the surface of the mf, the masking capacity, the variety of the antistress, detoxifying and antioxidants proteins, and antithrombotic capacity of the adult worms are mechanisms that contribute to the survival of the parasite [6, 26]. However, the cat is a less permissive host, with an intense proinflammatory immune response, which, on the one hand, can be lethal for itself and, on the other hand, impede or limit the survival of the adult worms and the production of mf [3]. Within wild reservoirs, the coyote, jackal, fox, wolf, and the raccoon dog can develop stable* D. immitis* and/or* D. repens* microfilarial infections, while other species only develop amicrofilaremic infections [15, 27, 28]. Given that the significance of a species as a reservoir is determined not only by the percentage of infected individuals but also by their survival and capacity to sustain the parasite long-term reproduction, the adaptations established between the dog and* D. immitis *and* D. repens* make this host an ideal reservoir. Among wild hosts, those that develop microfilaremic infections and show behavior that puts them in frequent contact with humans and pets environment, such as coyotes, foxes, jackals, or raccoon dogs, can be considered dangerous reservoirs [15, 29].

There are genetic differences both inter- and intraspecific that regulate the susceptibility and resistance of the mosquitoes to* Dirofilaria* [17, 30]. Furthermore, the invasion of the Malpighian tubules by the* Dirofilaria* larvae and their migration to the mouthparts is crucial for the survival of the mosquitoes. These have different structures and mechanisms that allow them to control the number of L3 larvae that complete their development. The cibarial armature, the coagulation of blood, the peritrophic membrane, the hemolymph defensins, and their melanization capacity eliminate part of developing larvae [17]. The percentage of infected vectors that survive the infection, the parasitic load that they are able to withstand, and the prevalence of infection determine the flow of L3 towards the vertebrate hosts.

*D. immitis* and* D. repens* coinfections in hosts and vectors have been described [15, 31]. However, there is little information regarding the interactions between both species coinciding within the same host. In experimental infections in dogs it was observed that when* D. repens* was the first species inoculated, its presence significantly decreased the number of* D. immitis* worms progressing to the adult stage when it was later introduced; this finding was not observed when the order of infection was reversed. This interaction, probably immune in nature, can influence the different patterns of prevalence observed [32]. Nonetheless, the fact that both species can simultaneously complete their life cycles in the same host suggests the existence of competitive exclusion, as it seems to occur in human filariae in Africa [33].

## 3. Epidemiology

**Figure 3:**
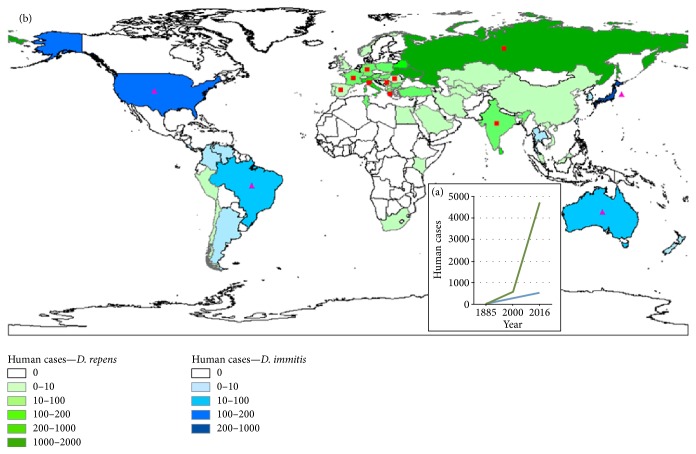
Changes in the incidence of human dirofilariasis reported cases (a). Geographic distribution of human dirofilariasis (reported cases) (b). Pulmonary dirofilariasis (blue); subcutaneous/ocular dirofilariasis (green); sporadic cases of subcutaneous/ocular dirofilariasis in areas where pulmonary dirofilariasis predominates (fuchsia triangles); sporadic cases of pulmonary dirofilariasis in areas where subcutaneous/ocular dirofilariasis predominates (red squares).

With respect to animal dirofilariasis, a great amount of the epidemiological information refers to canine dirofilariasis, while the information regarding domestic cats and wild reservoirs is, in general, limited. Human dirofilariasis is studied from two different perspectives, which have provided complementary information: seroepidemiological studies and the retrospective review of clinical cases previously published [34]. Various seroepidemiological studies have found significant seroprevalence of anti-*Dirofilaria* antibodies, which suggests a high risk of infection in human populations living in endemic areas [35–38]. The retrospective review of clinical cases highlights the actual incidence of the disease. Although it is widely accepted that in many countries human dirofilariasis is underdiagnosed, a dramatic increase in the level of incidence worldwide has been confirmed and is mainly subcutaneous/ocular in nature (Figure 3(a)) [6, 29, 39].

### 3.1. Geographic Distribution and Prevalence

#### 3.1.1. Dirofilariasis in the Animal Hosts

The episystem of dirofilariasis within Europe and Asia is characterized by the presence of* D. immitis* and* D. repens*, which are sympatric in most countries, while in a few only one of them has been reported [13] (Figure 2). The highest prevalence of* D. immitis* was found in the Canary Islands and Madeira and in Mediterranean countries (22–40%). Prevalence of* D. repens* ranged from 23 to 49% in Southwestern Russia and from 25% to 38% in some central and northern European countries [6, 40–42]. In Iran, China, and India, prevalence rates between 15 and 60% for both species have been reported [6, 43–47]. The prevalence of* D. immitis* has increased in some areas of India [45, 48] but in Japan has decreased from 46%, in 2001, to 23%, in 2010 [49].* D. immitis* has been found in feline populations in Portugal, Spain, and Italy, with prevalence rates between 3 and 27%, and there have been frequent reports in France [50–52]. Its presence has also been increasingly reported in European populations of foxes (3.7%–35%), jackals (7.7%–23.3%), and raccoon dogs (31.1%) and occasionally in wolves, while* D. repens* has been found in foxes, wolves, jackals, and badgers with prevalence rates that come close to 10% in some of the hosts [15, 53–55].

In the Americas* D. immitis* predominates, having been detected in the majority of the countries (Figure 2). The highest prevalence has been reported in the Eastern states of the USA, the Caribbean coast of Mexico, Caribbean Islands, and areas of Brazil and Argentina (20.4% to 74%) [6, 29, 56, 57]. Recent studies have described the notable increase of the prevalence in some Western states of USA [4], Mexico [58], Colombia [59], and Argentina [60]. Feline infections caused by* D. immitis* have been identified in canine endemic areas of the USA, Canada, Argentina, Brazil, and Venezuela. In the USA, the prevalence ranges between 3 and 19% and is higher in areas where canine prevalence is higher [61].* D. immitis* is also frequently detected in coyotes, foxes, and hybrids of both species and, occasionally, in other species [3, 62]. The prevalence in coyotes widely varies between 17% in Illinois [63] and 100% in Texas [62], and a spreading of the infection in the populations of California has been detected [27]. Also, infections in the maned wolf* (Chrysocyon brachyurus)* in Bolivia [64] and in the coati in Argentina [65] were reported. There is no data about the prevalence of* D. tenuis*,* D. ursi*,* D. subdermata*, and* D. striata* in wild hosts. Recently, the first* D. repens *infection in canines was reported in Mexico [66] and also one case in Chile, where the causal agent was genetically similar, but not identical, to* D. repens *[67].

*D. immitis* also predominates in the canine populations of Africa and Australia (Figure 2). In Africa, epidemiological information lacks in many countries. Prevalence of* D. immitis* is between 1% and 15% [6, 68, 69] and between 3 and 6% for* D. repens* [6, 70–72].* D. immitis* is endemic in the South Eastern Australia [73], where foxes in peri-urban areas and dingoes in areas with a low population density are the wild reservoirs [74, 75]. In New Zealand, infections caused by* D. immitis* and* D. repens* have been reported, probably being imported from Australia [76].

#### 3.1.2. Human Dirofilariasis

Human infections caused by* D. repens* widely predominate in Eurasia (Figure 3(b)), where approximately 4490 cases of subcutaneous/ocular dirofilariasis have been described. Of these, 4250 have occurred in Europe, with the highest incidences occurring in Ukraine (1934 cases), Russia (1440), Italy (326), and Belorussia (131), and with only 35 pulmonary cases attributed to* D. immitis* [6, 39, 77]. In Asia (Figure 3(b)), Sri Lanka, with 135 cases [78, 79], and India, with at least 100 subcutaneous/ocular cases and 3 pulmonary cases [80], are the countries with the highest level of incidence for human subcutaneous/ocular dirofilariasis. In other countries very few cases have been reported [43, 81–83]. Pulmonary dirofilariasis caused by* D. immitis* predominates in Japan, with 280 registered cases [5, 84–86] by only 3 subcutaneous cases [87].

In the Americas 175 cases of pulmonary infection in humans have been approximately reported, located in the USA (119 cases) [6, 88, 89] and Brazil (close to 50 cases) [6, 90], with sporadic reports in Costa Rica, Colombia, Venezuela, and Argentina [6] (Figure 3(b)). Only 34 cases of subcutaneous/ocular infection have been registered, of which 30 are from the USA and Canada, caused by* D. tenuis* [6, 91, 92],* D. ursi* or* D. subdermata *[6, 93, 94],* D. striata* [95],* Dirofilaria* spp. [96–98], and* D. immitis* [99]. Sporadic cases of subcutaneous and ocular infection have been reported in Chile, Peru, and Brazil [6, 90, 100].

Sixteen human cases have been recorded in Tunisia, 15 caused by* D. repens* and 1 caused by* D. immitis* [101], and other sporadic cases documented in South Africa and Egypt [102, 103]. In Australia 20 cases of pulmonary infection caused by* D. immitis* have been reported [6] and 1 case in New Zealand [104] (Figure 3(b)).

### 3.2. Climatic Change and Spreading of Dirofilariasis: Prediction Models

Industrial activity is modifying the climate, significantly increasing the temperature and global average precipitation with respect to the figures of the preindustrial age [105]. Vector-borne diseases are among the natural systems more sensitive to climate change, such as dirofilariasis, to be affected in different ways: increase in vector density, in the duration of their annual activity period and aggressiveness, the introduction of invasive species of competent vectors in endemic areas, and the shortening of the extrinsic incubation period of the parasite. Despite serious gaps within the information concerning the impact of climate change on the distribution and emergence/reemergence of pathogens and vectors and the fact that many of the results are debatable [106, 107], there are, however, studies that reasonably show a relationship between climate change and the alteration of the epidemiological situation. The average temperature recorded in Europe, within 2002 and 2011, is 1.3°C higher than during the preindustrial age [107]. Epidemiological studies have indicated that before 2000 dirofilariasis was almost exclusively associated with the southern European countries. Nevertheless, after this date dirofilariasis extended towards colder central and northern countries, with autochthonous cases having been reported in humans until a latitude of 61°N in Russia [6, 39]. In North America, where the temperature has increased between 1.3° and 1.9°F above those registered in 1895 [108], dirofilariasis has been gradually expanding over the decades [4, 16]. One recent study showed a significant relationship between the prevalence of canine dirofilariasis and temperature and precipitation, among other factors [109]. Exotic mosquito species, such as* Aedes albopictus* (the tiger mosquito), introduced in Europe and America through commercial activities, have rapidly expanded in many areas where dirofilariasis is endemic. One similar situation occurred with* Ae. koreicus* in Italy and Switzerland [110–112]. Both are competent vectors of* Dirofilaria* spp. with diurnal activity, complementing the afternoon or night activity of native species [17].

One of the fundamental objectives in the study of the relationship between climate and health is to create tools that allow the prediction of change, with the aim to prevent the outcomes. The integration of information obtained from numerous sources, such as geographic information systems (GIS), global positioning (GPS), remote sensing (RS) satellite systems, and epidemiological and climatic records, as well as the improvement of analysis software, has made achieving this objective possible [113]. With respect to dirofilariasis, the majority of the models published are based on the Growing Degree Days (GDD) concept, the accumulated heat needed to complete the extrinsic incubation of the* Dirofilaria* larvae in the life period of the vectors [114]. These models accurately predicted that the summer temperatures of nonendemic cold areas had reached a sufficient level for the extrinsic incubation of the larvae, allowing the calculation of the number of annual generations of* Dirofilaria*, and the length of the transmission period, at different geographic scales [7, 16, 114–116]. Additionally, they indicated the risk of introduction of* Ae. albopictus* and its hypothetical period of activity in nonendemic areas such as the UK [117, 118]. Other models incorporate local geo-environmental factors like the presence of irrigation in dry climate areas that refine predictions in Spain [20]. Recently, a complex hierarchical regression model relating multiple geo-climatic, social, and biological factors, as well as the prevalence of* D. immitis* in canine populations in the USA, county by county, has been created [4]. The reliability of the predications should be validated using real distribution and prevalence data. In the case of the USA, prevalence is one of the factors considered, thanks to the millions of diagnoses carried out in a standardized way throughout the country. In areas or countries where the diagnostic results do not permit validation, it can become complicated due to the lack or scarcity of epidemiological data, or validation is achieved “a posteriori” as new epidemiological data appear, years after the creation of the model. Despite that modelling is not an exact science, it has been accepted that the information generated could provide very valuable guidance for the application of programs aimed at controlling dirofilariasis [105].

## 4. Conclusions

Dirofilariasis is an extremely complex problem, primarily veterinary, but with an undoubted impact on human health and wildlife. In addition, in each continent there are biological and epidemiological peculiarities, which give each episystem its own characteristics. The progress of knowledge and management of dirofilariasis that has occurred primarily in the USA, Europe, and Japan has been possible thanks to a multidisciplinary approach to which parasitologists, veterinarians, doctors of different specialties, molecular biologists, computer scientists, mathematicians, and meteorologists have contributed. For this reason, dirofilariasis can be considered as a paradigm of the global health approach advocated by the one health concept. However, animal dirofilariasis continues to expand in many areas and cases of human dirofilariasis are reported with increasing frequency in more and more countries, while in others the disease is virtually unknown by specialists. In addition to future technical advances that will lead to the acquisition of more data, standardization of epidemiological surveillance procedures at the global level is key for the management improvements of dirofilariasis. The proven experience of societies such as the American Heartworm Society and more recently the European Society of Dirofilariasis and Angiostrongylosis, among others, can contribute to achieving the ultimate goal of effective global disease control.
